# Butylparaben Is Toxic to Porcine Oocyte Maturation and Subsequent Embryonic Development Following In Vitro Fertilization

**DOI:** 10.3390/ijms21103692

**Published:** 2020-05-24

**Authors:** Pil-Soo Jeong, Sanghoon Lee, Soo-Hyun Park, Min Ju Kim, Hyo-Gu Kang, Tsevelmaa Nanjidsuren, Hee-Chang Son, Bong-Seok Song, Deog-Bon Koo, Bo-Woong Sim, Sun-Uk Kim

**Affiliations:** 1 Futuristic Animal Resource & Research Center, Korea Research Institute of Bioscience and Biotechnology, Chungcheongbuk-do 28116, Korea; spectrum@kribb.re.kr (P.-S.J.); sodany2@kribb.re.kr (S.L.); tngusdl30@kribb.re.kr (S.-H.P.); jmmy05@kribb.re.kr (M.J.K.); kogd1887@kribb.re.kr (H.-G.K.); tsevelmaa@kribb.re.kr (T.N.); son1989@kribb.re.kr (H.-C.S.); sbs6401@kribb.re.kr (B.-S.S.); 2 Department of Biotechnology, Daegu University, Gyeongsangbuk-do 38453, Korea; dbkoo@daegu.ac.kr; 3 Department of Functional Genomics, KRIBB School of Bioscience, Korea University of Science and Technology (UST), Daejeon 34113, Korea

**Keywords:** butylparaben, porcine oocyte, in vitro maturation, ROS, mitochondria, apoptosis

## Abstract

Parabens are widely used in personal care products due to their antimicrobial effects. Although the toxicity of parabens has been reported, little information is available on the toxicity of butylparaben (BP) on oocyte maturation. Therefore, we investigated the effects of various concentrations of BP (0 μM, 100 μM, 200 μM, 300 μM, 400 μM, and 500 μM) on the in vitro maturation of porcine oocytes. BP supplementation at a concentration greater than 300 μM significantly reduced the proportion of complete cumulus cell expansion and metaphase II oocytes compared to the control. The 300 μM BP significantly decreased fertilization, cleavage, and blastocyst formation rates with lower total cell numbers and a higher rate of apoptosis in blastocysts compared to the control. The BP-treated oocytes showed significantly higher reactive oxygen species (ROS) levels, and lower glutathione (GSH) levels than the control. BP significantly increased the aberrant mitochondrial distribution and decreased mitochondrial function compared to the control. BP-treated oocytes exhibited significantly higher percentage of γ-H2AX, annexin V-positive oocytes and expression of LC3 than the control. In conclusion, we demonstrated that BP impaired oocyte maturation and subsequent embryonic development, by inducing ROS generation and reducing GSH levels. Furthermore, BP disrupted mitochondrial function and triggered DNA damage, early apoptosis, and autophagy in oocytes.

## 1. Introduction

Parabens, a family of alkyl esters of para-hydroxybenzoic acid, are classified based on the alkyl chain as methylparaben, ethylparaben, propylparaben, benzylparaben, or butylparaben (BP) [1]. Their antimicrobial effects disrupt the membrane transporter system or inhibit ATPases and phosphotransferases, and as a result of this, parabens are widely used in cosmetics, foods, and pharmaceuticals [2,3]. The antimicrobial effects of paraben increases as the alkyl chain length of the ester group increases [4]. However, the greater the length of the alkyl chain, the higher the lipid solubility, which leads to a high rate of penetration of parabens through the epidermis [5]. Humans are mainly exposed to parabens through skin absorption and ingestion of personal care products, but parabens have also been detected in the natural environment, including in indoor dust, the surface water of rivers, sediment, and sludge [6]. Other studies have reported parabens in human tissue and urine [7,8]. Although parabens are considered safe at certain concentrations, continuous exposure may pose a potential threat to human health [9]. However, overall, little is known about the possible effects of parabens on human health.

Parabens bind to estrogen receptors, because they are structurally similar to estrogen [10]. BP has the most potent estrogenic activity among the parabens [11]. BP is one of the most common preservatives in consumer products, because of its low toxicity and antimicrobial properties [12]. However, several studies have shown that BP affects the reproductive system by interfering with hormone functioning. Previous studies reported that BP exposure exerted adverse effects on sperm production, the capacity for fertilization, and secretion of testosterone in male rats [13,14]. In the female reproductive system, BP exposure changed the uterine histomorphological structure in ovariectomized mice [15]. Moreover, BP exposure disrupted hormone responses and the morphology of follicles in peripubertal rats [16], and inhibited early phase folliculogenesis in neonatal rats [17].

Although several studies have reported the effects of BP exposure on the male and female reproductive system, little is known regarding the effects of BP on oocyte maturation. Moreover, rodents have many anatomical and physiological differences to humans [18]. Therefore, in this study, we investigated the toxicity of BP on oocyte maturation and subsequent embryo development following fertilization using porcine oocytes, which have genetic and physiological characteristics similar to those of humans [19]. We investigated the effects of BP during in vitro maturation (IVM) on cumulus cell expansion, oocyte nuclear maturation, and the developmental competence of embryos following in vitro fertilization (IVF). In addition, we measured intracellular reactive oxygen species (ROS) and glutathione (GSH) levels, and mitochondrial distribution and function, and confirmed early apoptosis and DNA damage in porcine oocytes after BP exposure.

## 2. Results

### 2.1. BP Treatment Impairs Meiotic Progression in Porcine Oocytes

**Figure 1:**
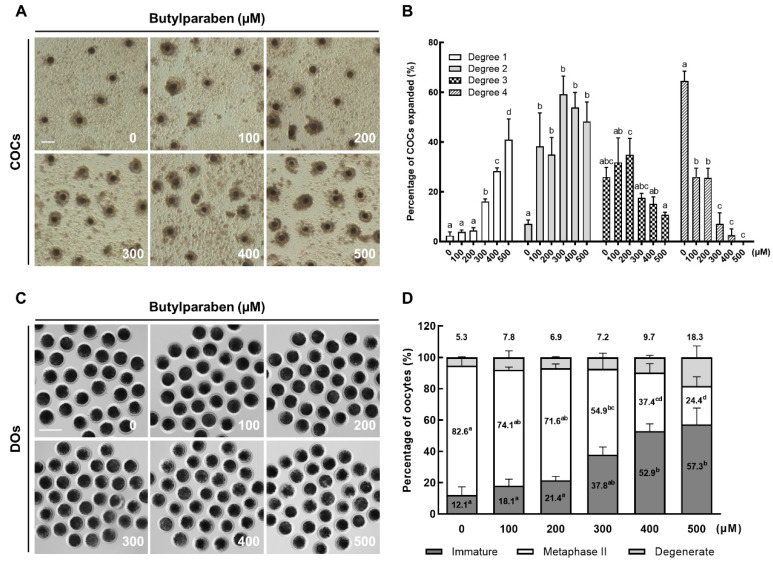
Effects of butylparaben (BP) exposure on porcine oocyte maturation. (**A**) Representative photographs and (**B**) the percentage of cumulus cell expansion in the control and BP-treated groups after 44 h of in vitro maturation (IVM) (*n* = 131 per group). (**C**) Representative photographs of in vitro matured oocytes and (**D**) the percentage of different stages of nuclear maturation in the control and BP-treated groups after 44 h of IVM (*n* = 131 per group). Bar = 200 μm. The data are from three independent experiments and the values represent the mean ± SEM. Values with different superscript letters (a–d) differ significantly (*p* < 0.05). BP, butylparaben; COCs, cumulus-oocyte complexes; DOs, denuded oocytes.

To evaluate the effect of BP on meiotic progression in porcine oocytes, we cultured cumulus-oocyte complexes (COCs) in maturation medium supplemented with different concentrations of BP (0 μM, 100 μM, 200 μM, 300 μM, 400 μM, and 500 μM) during IVM, and investigated the cumulus cell expansion and oocyte nuclear maturation. BP treatment significantly decreased the proportion of COCs exhibiting complete cumulus expansion (degree 4; the maximum degree of expansion, including the corona radiata) in a dose-dependent manner. The 500 μM BP group showed a significantly lower proportion of degree 3 (all cell layers except the corona radiata have expanded), compared to the 200 μM BP group. The proportion of degree 2 (only the outermost layers of cumulus cells have expanded) was significantly increased in all BP-treated groups, and the proportion of degree 1 (the minimum observable response, with spherical and compacted cumulus cells seen around the oocyte) was significantly increased in 300, 400, and 500 μM BP-treated groups, compared to the control (Figure 1A,B). Moreover, BP treatment decreased the proportion of metaphase II (MII) oocytes, and increased that of immature oocytes, in a dose-dependent manner. In particular, 300, 400, and 500 μM BP-treated groups showed significantly lower proportions of MII compared to the control (Figure 1C,D). In the following experiments, 300 μM BP was used.

### 2.2. BP Treatment during IVM Reduces the Developmental Competence of Porcine IVF Embryos

**Figure 2:**
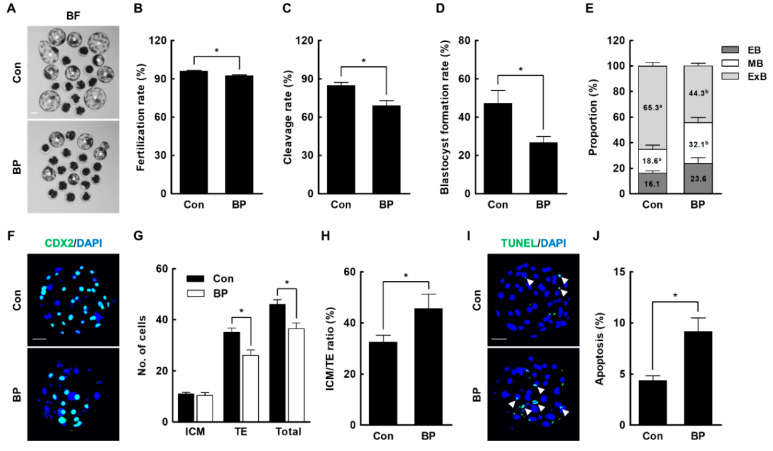
Effects of butylparaben (BP) exposure during in vitro maturation (IVM) on embryonic development after in vitro fertilization (IVF). (**A**) Representative photographs of blastocysts developed from control and BP-treated oocytes after IVF. Bar = 100 μm. (**B**) Fertilization rates (**C**), cleavage rates, and (**D**) blastocyst formation rates of embryos from control and BP-treated oocytes after IVF (Con; *n* = 181, BP; *n* = 118). (**E**) Proportion of blastocyst stages of control and BP-treated oocytes after IVF (Con; *n* = 79, BP; *n* = 29). (**F**) Representative immunofluorescence photographs of CDX2/DAPI using blastocysts developed in the BP-treated and control groups. Merged images (light green) between DAPI (blue) and CDX2 (green) signals are shown. Bar = 50 μM. Quantification of (**G**) the inner cell mass (ICM), trophectoderm cells (TE), total cell numbers, and (**H**) ICM/TE ratios in the BP-treated and control groups (Con; *n* = 20, BP; *n* = 20). (**I**) Representative immunofluorescence photographs of terminal deoxynucleotidyl transferase-mediated dUTP-digoxygenin nick end-labeling (TUNEL) assay using blastocysts developed in the BP-treated and control groups. Merged images (light green) between DAPI (blue) and TUNEL (green, white arrow) signals are shown. Bar = 50 μM. (**J**) Quantification of proportion of apoptotic cells in the BP-treated and control groups (Con; *n* = 21, BP; *n* = 21). The data are from three independent experiments and the values represent the mean ± SEM. * *p* < 0.05. Values with different superscript letters (a and b) differ significantly (*p* < 0.05). EB, early blastocyst; MB, middle blastocyst; ExB, expanded blastocyst.

Next, we investigated the fertilization and subsequent embryonic development of BP-treated MII oocytes following IVF. BP-treated MII oocytes showed a significantly decreased fertilization rate (Con, 96.0 ± 0.7% vs. BP, 92.4 ± 0.6%), cleavage rate (Con, 84.7 ± 2.5% vs. BP, 68.9 ± 4.0%), and blastocyst formation rate (Con, 47.2 ± 6.7% vs. BP, 26.7 ± 3.2%) compared to the control (Figure 2A–D). Development during the post-blastulation period was significantly delayed in the BP-treated group (Figure 2E). In addition, we further assessed the quality of blastocysts using CDX2 staining and the terminal deoxynucleotidyl transferase-mediated dUTP-digoxygenin nick end-labeling (TUNEL) assay. Although there was no difference in the number of cells in the inner cell mass (ICM) (Con, 10.9 ± 0.8 vs. BP, 10.5 ± 1.0) between the control and BP-treated group, the BP-treated group showed significantly reduced trophectoderm (TE) (Con, 35.0 ± 1.7 vs. BP, 26.1 ± 2.1) and total cell numbers (Con, 46.0 ± 1.8 vs. BP, 36.5 ± 2.3), leading to an abnormally increased ICM cell to TE cell ratio (Con, 32.5 ± 2.8% vs. BP, 45.7 ± 5.6%) in the BP-treated group (Figure 2F–H). Moreover, the BP-treated group exhibited significantly increased cellular apoptosis levels compared to the control (Con, 4.4 ± 0.5% vs. BP, 9.2 ± 1.3%) (Figure 2I,J). These results indicated that BP exposure during IVM impaired oocyte maturation, and thereby led to reduced subsequent embryo development after IVF.

### 2.3. BP Treatment Negatively Affects Intracellular Levels of ROS and GSH in Porcine Oocytes

**Figure 3:**
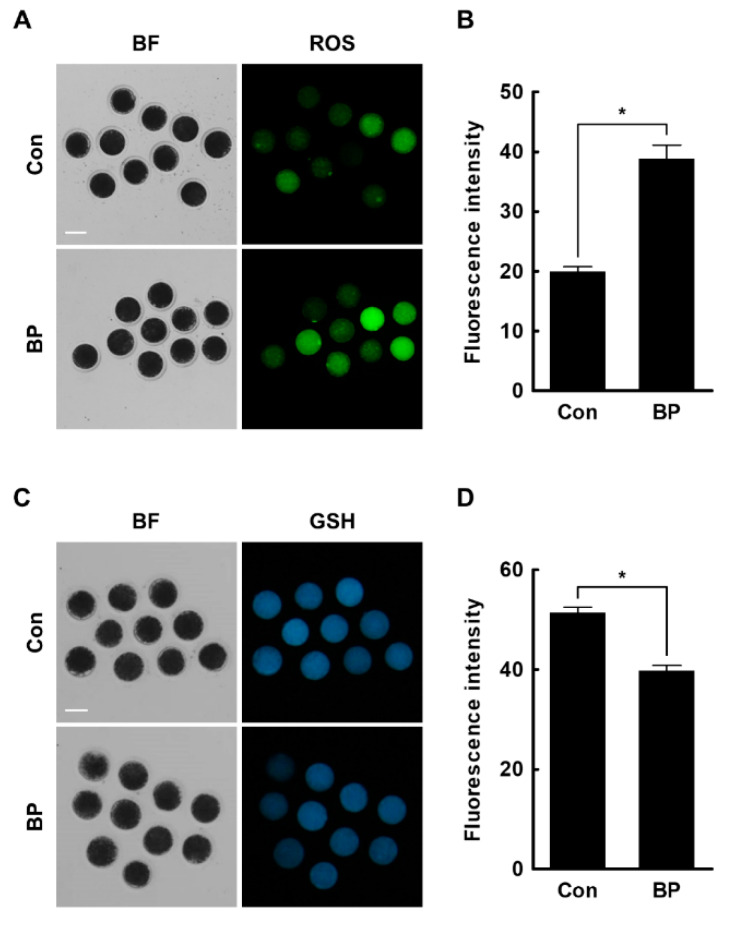
Effects of butylparaben (BP) exposure during in vitro maturation (IVM) on intracellular reactive oxygen species (ROS) and glutathione (GSH) levels. (**A**) Fluorescence images of oocytes treated with CM-H2DCFDA for measurement of intracellular ROS levels in the BP-treated and control groups. Bar = 100 μM. (**B**) Quantification of the fluorescence intensity in the BP-treated and control groups (Con; *n* = 50, BP; *n* = 50). (**C**) Fluorescence images of oocytes treated with CMF2HC for measurement of the intracellular GSH level in the BP-treated and control groups. Bar = 100 μM. (**D**) Quantification of the fluorescence intensity in the BP-treated and control groups (Con; *n* = 50, BP; *n* = 50). The data are from three independent experiments and the values represent the mean ± SEM. * *p* < 0.05.

To determine how BP affected the developmental competence of porcine oocytes, we examined the intracellular ROS and GSH levels in BP-treated oocytes. The ROS levels were significantly higher in the BP-treated group compared to the control (Con, 20.3 ± 1.0 vs. BP, 42.2 ± 3.2) (Figure 3A,B). Consistent with these results, GSH levels were significantly lower in the BP-treated group compared to the control (Con, 51.5 ± 1.0 vs. BP, 39.7 ± 1.2) (Figure 3C,D).

### 2.4. BP Treatment Interferes with Mitochondrial Organization and Function in Porcine Oocytes

**Figure 4:**
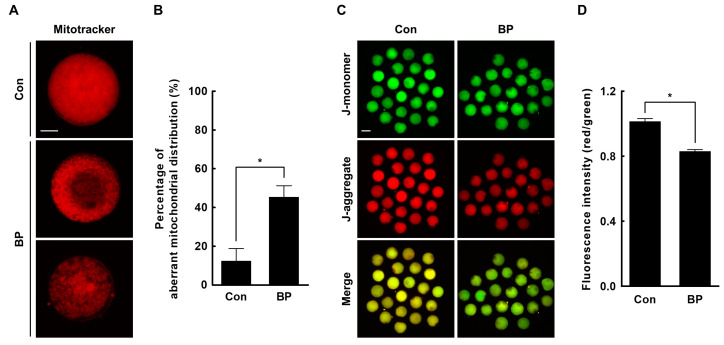
Effects of butylparaben (BP) exposure during in vitro maturation (IVM) on mitochondrial distribution and function. (**A**) Fluorescence images of oocytes stained with MitoTracker Deep Red in the BP-treated and control groups. Bar = 50 μM. (**B**) Percentage of oocytes with aberrant mitochondrial distribution in the BP-treated and control groups (Con; *n* = 53, BP; *n* = 53). (**C**) Fluorescence images of oocytes stained with JC-1 in the BP-treated and control groups. Bar = 100 μM. (**D**) Quantification of the fluorescence intensity (red/green) in the indicated groups (Con; *n* = 62, BP; *n* = 62). The data are from three independent experiments and the values represent the mean ± SEM. * *p* < 0.05.

The mitochondrion is an important organelle that produces cellular energy through oxidative metabolism during oocyte maturation, fertilization, and embryonic development. Therefore, mitochondrial distribution and activity are critical indicators of oocyte cytoplasmic maturation [20]. We evaluated mitochondrial distribution and membrane potential in BP-treated oocytes using MitoTracker and JC-1 staining, respectively. The rate of aberrant mitochondrial distribution, including a semi-peripheral distribution, was significantly higher in the BP-treated group (Con, 12.4 ± 6.3% vs. BP, 45.4 ± 5.8%); most oocytes in the control group showed a homogeneous mitochondrial distribution (Figure 4A,B). Furthermore, BP-treated oocytes showed a significantly reduced J-aggregate (high membrane potential)/J-monomer (low membrane potential) ratio, compared to the control (Con, 1.0 ± 0.0 vs. BP, 0.8 ± 0.0) (Figure 4C,D), suggesting that BP exposure negatively affected mitochondrial organization and function during oocyte maturation.

### 2.5. BP Treatment Triggers DNA Damage, Early Apoptosis, and Autophagy in Porcine Oocytes

**Figure 5:**
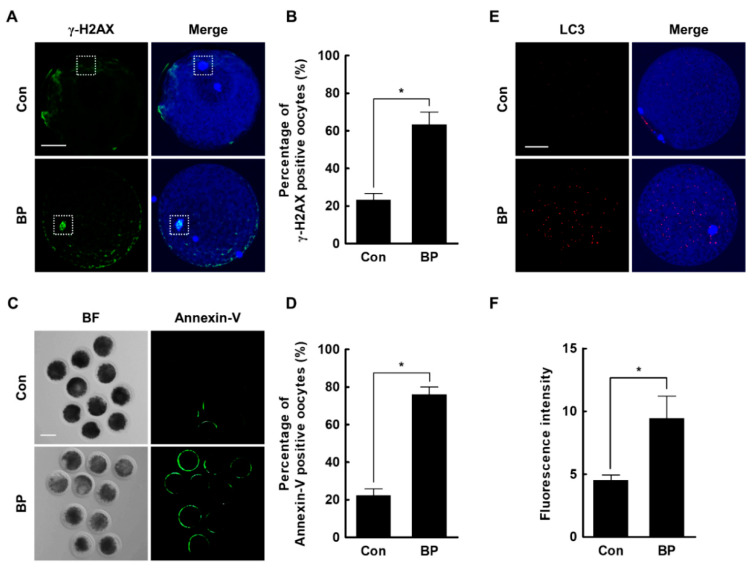
Effects of butylparaben (BP) exposure during in vitro maturation (IVM) on DNA damage, early apoptosis, and autophagy. (**A**) Fluorescence images of oocytes stained with an antibody against γ-H2AX in the BP-treated and control groups. Bar = 50 μM. (**B**) Percentage of γ-H2AX-positive oocytes in the BP-treated and control groups (Con; *n* = 30, BP; *n* = 30). (**C**) Fluorescence images of oocytes stained with annexin V in the BP-treated and control groups. Bar = 100 μM. (**D**) Percentage of annexin V-positive oocytes in the BP-treated and control groups (Con; *n* = 50, BP; *n* = 50). (**E**) Fluorescence images of oocytes stained with an antibody directed against LC3 in the BP-treated and control groups. Bar = 50 μM. (**F**) Quantification of the fluorescence intensity in the BP-treated and control groups (Con; *n* = 26, BP; *n* = 26). The data are from three independent experiments and the values represent the mean ± SEM. * *p* < 0.05.

Finally, to determine the toxic effects of BP in porcine oocytes, we investigated DNA damage, early apoptosis, and autophagy in BP-treated oocytes. We found that the rate of γ-H2AX-positive oocytes was significantly higher in the BP-treated group compared to the control (Con, 23.3 ± 3.3% vs. BP, 63.3 ± 6.7%) (Figure 5A,B). Moreover, the BP-treated oocytes showed a significantly higher proportion of annexin V-positive oocytes (Con, 22.2 ± 3.6% vs. BP, 76.0 ± 4.0%), and higher microtubule-associated protein light chain 3 (LC3) levels, compared to the control (Con, 4.5 ± 0.4 vs. BP, 9.4 ± 1.8) (Figure 5C–F). Collectively, these results indicated that BP exposure during porcine oocyte maturation caused DNA damage and induced cell death, including early apoptosis and autophagy.

## 3. Discussion

Parabens exist in the natural environment, including in indoor dust, the surface water of rivers, and soil. In addition, many people are exposed to parabens through the skin, because they are used as additives in personal care products, cosmetics, and foods. Several studies reported that parabens (in particular BP, detected in human tissues and urine) have adverse effects on human health [21]. Previous studies reported that parabens negatively affected the female reproductive system by decreasing ovarian weight, follicle development, and pregnant maintenance [22]. Moreover, exposure to isobutylparaben during IVM disrupts oocyte maturation through cytoskeleton, ROS, and epigenetic modifications [23]. As BP has the most potent estrogenic effects among parabens, exposure to BP could be more harmful to the female reproductive system. However, the toxic effects of BP on oocyte meiotic progression have not been studied. In the present study, we demonstrated that BP exposure negatively affected cumulus cell expansion, oocyte nuclear maturation, and subsequent embryonic development. Moreover, BP exposure negatively affected the regulation of intracellular ROS and GSH levels, and mitochondrial function, leading to DNA damage, early apoptosis, and autophagy in porcine oocytes.

In general, cumulus cells surrounding the oocyte are required for oocyte maturation during meiotic progression; cumulus cells communicate with one another, and with the oocyte, via gap junctions, thus providing signals for oocyte maturation [24]. Furthermore, cumulus cell expansion contributes to the developmental competence of oocytes and subsequent embryonic development [25]. Therefore, cumulus cell expansion is an important indicator of oocyte maturation and fertilization ability [26]. In this study, we showed that BP exposure during IVM significantly decreased cumulus cell expansion and the proportion of MII oocytes in a dose-dependent manner, indicating that BP exposure causes the failure of porcine oocyte maturation. Moreover, BP-treated MII oocytes exhibited significantly lower fertilization ability and developmental competence of IVF embryos. These results indicate that BP impairs meiotic progression of porcine oocytes through the inhibition of cumulus cell expansion and oocyte nuclear maturation, thereby impairing embryonic development.

Intracellular ROS and GSH levels are crucial factors in oocyte maturation and subsequent embryo development following fertilization [27]. ROS are produced as a byproduct of cellular energy metabolism, and excessive accumulation of ROS can induce cellular damage and apoptosis, which may lead to meiotic arrest and impaired embryonic development [28]. GSH is involved in various physiological processes, including amino acid transfer and protein synthesis [29], and also exerts antioxidant effects by protecting cells from oxidative stress [30]. Moreover, the GSH level in the oocyte reflects cytoplasmic maturation [31]. Previous studies reported that BP exerts oxidative stress by reducing the levels of non-enzymatic antioxidants, such as GSH, and the levels of enzymatic antioxidants, such as superoxide dismutase, catalase, and GSH transferase, which results in cellular apoptosis [32,33,34]. In this study, BP-treated oocytes showed significantly increased ROS levels and decreased GSH levels, indicating that BP induced oxidative stress during oocyte maturation. In addition, ROS can disrupt mitochondrial organization, membrane potential, and ATP production [35,36]. Well-organized mitochondria with a homogenous distribution in the cytoplasm and high mitochondrial membrane potential are considered important for oocyte cytoplasmic maturation [20]. A previous study reported that BP had toxic effects on human trophoblast cells through oxidative stress-induced mitochondrial dysfunction [33]. In this study, BP-treated oocytes exhibited significantly higher aberrant mitochondrial distribution and had a significantly lower mitochondrial membrane potential, indicating that BP compromised mitochondrial function and cytoplasmic maturation. Taken together, these results indicate that BP exposure has detrimental effects during porcine oocyte maturation, leading to impaired embryonic development.

ROS-related oxidative stress can reduce oocyte quality and fertilization ability by inducing DNA damage [37]. Γ-H2AX has been used as a biomarker for DNA damage caused by cytotoxic chemical agents [38]. Previous studies reported that BP induced DNA damage in rats and humans [9,39]. Consistent with previous studies, our results demonstrated that the proportion of γ-H2AX-positive oocytes was significantly higher in the BP-treated group than the control group, indicating that BP exposure may attenuate oocyte maturation by disrupting DNA integrity. In addition, oxidative stress is associated with cell death via apoptosis and autophagy. Excessive accumulation of ROS induces cellular apoptosis and reduces oocyte quality during meiotic progression [40]. Previously, early apoptosis was detected by annexin V, a phospholipid-binding protein that binds to phosphatidylserine [41]. We found that BP-treated oocytes showed significantly higher rates of early apoptosis. Moreover, it has been reported that excessive accumulation of ROS can induce autophagy, which is an important cellular event during oocyte maturation [42], where high autophagic activity negatively affects mitochondrial function during oocyte maturation [43]. We found that LC3 levels were significantly higher in BP-treated oocytes compared to the control, indicating that BP exposure might induce autophagy during oocyte maturation. Collectively, these results indicated that BP-induced ROS accumulation might cause DNA damage, early apoptosis, and autophagy. Therefore, oxidative stress-induced DNA damage could be the specific mechanism by which BP impaired oocyte maturation and further embryonic development.

In conclusion, to the best of our knowledge, this is the first study demonstrating the toxicity of BP on oocyte maturation. These findings provide evidence that BP exposure during meiotic progression impairs oocyte quality and subsequent embryonic development following IVF, by inducing ROS generation and reducing GSH levels. Moreover, BP-induced ROS disrupted mitochondrial function and triggered DNA damage, early apoptosis, and autophagy. These findings provide an opportunity to raise awareness of the toxicity of BP on oocyte maturation, and to elucidate how BP detrimentally affects oocyte maturation.

## 4. Materials and Methods

### 4.1. Chemicals

Unless stated otherwise, all chemicals and reagents used in the present study were purchased from Sigma-Aldrich Chemical Company (St. Louis, MO, USA).

### 4.2. Oocyte Collection and IVM

Porcine ovaries were obtained at a local slaughterhouse and transported to the laboratory in 0.9% saline containing streptomycin sulfate (50 μg/mL) and potassium penicillin G (75 μg/mL) at 38.5 °C within 2 h. COCs were aspirated from 3 to 7 mm antral follicles using an 18-gauge needle attached to a disposable 10 mL syringe, and were washed three times with Tyrode’s albumin lactate pyruvate-HEPES medium. Next, approximately 40–50 COCs were transferred in 500 μL IVM medium in a four-well multidish (Nunc, Roskilde, Denmark) for 22 h at 38.5 °C in 5% CO_2_ in air. The IVM medium consisted of tissue culture medium-199 containing 10% porcine follicular fluid, 10 IU/mL pregnant mare serum gonadotropin (PMSG), and 10 IU/mL human chorionic gonadotropin (hCG), 0.57 mM cysteine, 10 ng/mL β-mercaptoethanol, and 10 ng/mL epidermal growth factor. After cultivation for 22 h, the COCs were further cultivated to hormone-free IVM medium for additional 22 h at 38.5 °C in 5% CO_2_ in air.

### 4.3. Treatment of Butylparaben

Butylparaben was dissolved in dimethylsulfoxide (DMSO), and then diluted with IVM medium to final concentrations of 100 μM, 200 μM, 300 μM, 400 μM, and 500 μM, with 0.2% DMSO in the IVM medium.

### 4.4. Assessment of Cumulus Cell Expansion

Assessment of the cumulus cell expansion performed as described previously [44,45]. After 44 h of IVM, the degree of cumulus cell expansion in porcine COCs was assessed by morphological examination under a microscope. Briefly, a degree of 0 indicates no expansion, and a degree of 1 indicates the minimum observable response, with spherical and compacted cumulus cells seen around the oocyte. A degree of 2 indicates that only the outermost layers of cumulus cells have expanded. A degree of 3 indicates that cumulus cell layers except the corona radiata have expanded, and a degree of 4 indicates the maximum degree of cumulus cells expansion, including the corona radiate.

### 4.5. Assessment of Nuclear Maturation of Oocytes

After 44 h of IVM culture, cumulus cells were removed by gently pipetting with 0.1% hyaluronidase in Dulbecco’s phosphate-buffered saline (DPBS; Gibco, Grand Island, NY, USA) supplemented with 4 mg/mL bovine serum albumin (BSA) (PB1) medium. The denuded oocytes were classified as immature (without first polar body extrusion), degenerate, or MII under a microscope (Nikon Corp., Tokyo, Japan) [46].

### 4.6. IVF of Oocytes

IVF was performed as described previously [47]. MII oocytes were rinsed three times with modified Tris-buffered medium (mTBM) containing 2.5 mM caffeine sodium benzoate and 1 mg/mL BSA. Then, 10–15 oocytes were transferred into 48 µL droplets of IVF medium under mineral oil pre-equilibrated at 38.5 °C in 5% CO_2_ in air. For the preparation of spermatozoa using the swim-up method, semen samples were washed three times with sperm washing medium (DPBS; Gibco, Invitrogen, Carlsbad, CA, USA) containing 1 mg/mL BSA, 100 µg/mL penicillin G, and 75 µg/mL streptomycin sulfate. After washing, 2 mL of sperm washing medium was gently added to the spermatozoa pellet and incubated for 15 min at 38.5 °C in 5% CO_2_ in air. After incubation, the supernatant was resuspended with 1 mL of mTBM. Next, 2 µL of diluted spermatozoa was added to a 48 µL droplet of mTBM to a final concentration of 1.5 × 10^5^ spermatozoa/mL. After 6 h of co-incubation, the loosely attached sperm were stripped by gentle pipetting and embryos were cultured in 40 μL droplets of porcine zygote medium-3 at 38.5 °C in 5% CO_2_ in air for 6 days. The number of embryos cleaved and developed to blastocysts stage was scored at 2 and 6 days of in vitro culture, respectively. Blastocysts were classified into three grades by morphological status. Early blastocyst indicates small size blastocyst with a blastocoel less than half of the embryo volume, middle blastocyst indicates middle size blastocyst with a blastocoel greater than half of the embryo volume, and expanded blastocyst indicates large size blastocyst with a blastocoel completely filling the embryo volume.

### 4.7. Terminal Deoxynucleotidyl Transferase-Mediated dUTP-Digoxygenin Nick End-Labeling (TUNEL) Assay

To count apoptotic cells in the blastocysts, TUNEL assay was carried out using an in situ cell death detection kit (Roche, Basel, Switzerland). Blastocysts were washed three times with DPBS supplemented with 1 mg/mL polyvinyl alcohol (PVA) (DPBS-PVA), and fixed in 4% paraformaldehyde overnight at 4 °C. For permeabilization, fixed blastocysts were incubated in DPBS containing 0.5% (*v*/*v*) Triton X-100 at room temperature (RT) for 30 min and then washed three times in DPBS-PVA and incubated with fluorescein-conjugated dUTP and terminal deoxynucleotidyl transferase for 1 h at 38.5 °C. Subsequently, the blastocysts were washed three times in DPBS-PVA and mounted on slide glasses with Vectashield containing DAPI (Vector Laboratories, Burlingame, CA, USA). DAPI-labeled or TUNEL-positive nuclei were observed with a fluorescence microscope (DMi8; Leica Microsystems, Wetzlar, Germany).

### 4.8. Measurement of Intracellular ROS and GSH Levels

Intracellular ROS and GSH levels were detected by CM-H2DCFDA (Invitrogen, Carlsbad, CA, USA) and CMF2HC (Invitrogen), respectively. The oocytes were washed three times with DPBS-PVA and incubated in DPBS-PVA containing 5 µM CM-H2DCFDA or CMF2HC for 30 min. After incubation, oocytes were washed three times with DPBS-PVA, and fluorescence was observed under a fluorescence microscope (DMi8; Leica Microsystems, Wetzlar, Germany) with ultraviolet filters (460 nm for ROS and 370 nm for GSH). The fluorescence intensity was analyzed using ImageJ software (version 1.47; National Institutes of Health, Bethesda, MD, USA) after normalization through subtraction of the background intensity from each oocyte size. Ten to fifteen oocytes were used for each detection, and the experiment was replicated three times.

### 4.9. Assessment of Mitochondrial Distribution and Membrane Potential

To determine the distribution of active mitochondria and mitochondrial membrane potential, oocytes were stained after 44 h of IVM. Oocytes were incubated in DPBS-PVA containing 0.5 μM MitoTracker Deep Red FM (Invitrogen) or JC-1 (1:100) (Cayman Chemical, Ann Arbor, MI, USA) for 30 min at 39 °C in 5% CO_2_ in air. Labeled oocytes were then washed three times in DPBS-PVA, for 10 min each wash. After washing, samples were fixed in 4% paraformaldehyde for 2 h at RT. After fixed samples had been washed three times for 10 min each in DPBS-PVA, oocytes were immediately observed under a fluorescence microscope (DMi8; Leica Microsystems) for JC-1 staining, or were mounted on slide glasses in Vectashield containing DAPI (Vector Laboratories, Burlingame, CA, USA), and observed under a fluorescence microscope for MitoTracker staining (Invitrogen, Carlsbad, CA, USA). Fluorescence was quantified using ImageJ software after normalization, through subtraction of the background intensity from each oocyte size. Twenty to twenty-five oocytes were used for each detection, and the experiment was replicated three times.

### 4.10. Annexin-V Staining

To detect the early apoptosis, oocytes were stained using the FITC Annexin-V Apoptosis Detection Kit (#556547; BD biosciences, Franklin Lake, NJ, USA). The oocytes were washed three times in DPBS-PVA and then incubated in 100 μL binding buffer containing 5 μL of Annexin-V-FITC for 30 min at 38.5 °C in the dark. The oocytes were determined immediately with a laser-scanning confocal fluorescent microscope (LSM700; Zeiss, Oberkochen, Germany).

### 4.11. Immunocytochemistry

Oocytes or blastocysts were washed three times in DPBS-PVA and fixed in 4% paraformaldehyde overnight at 4 °C. For permeabilization, fixed oocytes or blastocysts were treated with DPBS containing 1% Triton X-100 for 1 h at RT, and then washed three times in DPBS-PVA. Next, the oocytes or blastocysts were blocked with DPBS-PVA supplemented with 1 mg/mL BSA (DPBS-PVA-BSA) at 4 °C overnight. For CDX2 staining, blastocysts were additionally stored with 10% normal goat serum for 1 h at RT. After blocking, oocytes or blastocysts were incubated in primary antibodies, rabbit polyclonal anti-LC3B (1:100; #2775; Cell Signaling Technology, Beverly, MA, USA) and rabbit anti-Phospho-Histone H2A.X (Ser139) (1:200; #2577; Cell Signaling Technology) for oocytes, and mouse monoclonal anti-CDX2 (an undiluted solution; BioGenex Laboratories Inc., San Ramon, CA, USA) for blastocysts, at 4 °C for overnight. After washing three times with DPBS-PVA-BSA for 10 min each wash, oocytes or blastocysts were incubated for 1 h at RT with conjugated secondary antibodies, Alexa Fluor 488-labeled goat anti-rabbit or anti-mouse IgG (1:200). After washing three times in DPBS-PVA-BSA for 10 min, oocytes or blastocysts were mounted on slide glasses in Vectashield containing DAPI (Vector Laboratories) and observed under a fluorescence microscope (DMi8; Leica Microsystems, Wetzlar, Germany).

### 4.12. Statistical Analysis

Data are expressed as the mean ± standard error of the mean (SEM). Each experiment was replicated at least three times. Data were analyzed by analysis of variance (ANOVA), followed by Tukey’s multiple range test (Figure 1; data on cumulus cells expansion and oocyte nuclear maturation) or Student’s t-test (Figure 2; data on development of IVF embryos, Figure 3; data on ROS and GSH levels, Figure 4; data on mitochondrial distribution and membrane potential, Figure 5; data on γ-H2AX, LC3, and annexin-V), using SigmaStat Software (SPSS Inc., Chicago, IL, USA). *p*-values less than 0.05 were considered statistically significant.

## Abbreviations

BP	butylparaben
ROS	reactive oxygen species
GSH	glutathione
IVM	in vitro maturation
IVF	in vitro fertilization
COCs	Cumulus-oocyte complexes
MII	metaphase II
ICM	inner cell mass
TE	trophectoderm
TUNEL	Terminal deoxynucleotidyl transferase-mediated dUTP-digoxygenin nick end-labeling
LC3	microtubule-associated protein light chain 3
PMSG	pregnant mare serum gonadotropin
hCG	human chorionic gonadotropin
DMSO	dimethylsulfoxide
DPBS	Dulbecco’s phosphate-buffered saline
BSA	bovine serum albumin
mTBM	modified Tris-buffered medium
PVA	polyvinyl alcohol
RT	room temperature
SEM	standard error of the mean
ANOVA	analysis of variance
